# Improving Yield Components and Desirable Eating Quality of Two Wheat Genotypes Using Si and NanoSi Particles under Heat Stress

**DOI:** 10.3390/plants11141819

**Published:** 2022-07-11

**Authors:** Nesma M. Helal, Hemmat I. Khattab, Manal M. Emam, Gniewko Niedbała, Tomasz Wojciechowski, Inès Hammami, Nadiyah M. Alabdallah, Doaa Bahaa Eldin Darwish, Mohamed M. El-Mogy, Heba M. Hassan

**Affiliations:** 1 Botany Department, Faculty of Science, Ain Shams University, Cairo 11566, Egypt; nesmaflax@yahoo.co.uk (N.M.H.); dr.hemmat@hotmail.com (H.I.K.); emammanal@ymail.com (M.M.E.); 2 Department of Biosystems Engineering, Faculty of Environmental and Mechanical Engineering, Poznań University of Life Sciences, Wojska Polskiego 50, 60-627 Poznań, Poland; tomasz.wojciechowski@up.poznan.pl; 3 Department of Biology, College of Science, Imam Abdulrahman Bin Faisal University, P.O. Box 1982, Dammam 31441, Saudi Arabia; ihammami@iau.edu.sa (I.H.); nmalabdallah@iau.edu.sa (N.M.A.); 4 Botany Department, Faculty of Science, Mansoura University, Mansoura 35511, Egypt; ddarwish@ut.edu.sa; 5 Biology Department, Faculty of Science, University of Tabuk, Tabuk 46429, Saudi Arabia; 6 Vegetable Crops Department, Faculty of Agriculture, Cairo University, Giza 12613, Egypt; elmogy@agr.cu.edu.eg

**Keywords:** wheat, silicon, silicon nanoparticles, heat stress, late sowing, yield components

## Abstract

Global climate change is a significant challenge that will significantly lower crop yield and staple grain quality. The present investigation was conducted to assess the effects of the foliar application of either Si (1.5 mM) or Si nanoparticles (1.66 mM) on the yield and grain quality attributes of two wheat genotypes (*Triticum aestivum* L.), cv. Shandweel 1 and cv. Gemmeiza 9, planted at normal sowing date and late sowing date (heat stress). Si and Si nanoparticles markedly mitigated the observed decline in yield and reduced the heat stress intensity index value at late sowing dates, and improved yield quality via the decreased level of protein, particularly glutenin, as well as the lowered activity of α-amylase in wheat grains, which is considered a step in improving grain quality. Moreover, Si and nanoSi significantly increased the oil absorption capacity (OAC) of the flour of stressed wheat grains. In addition, both silicon and nanosilicon provoked an increase in cellulose, pectin, total phenols, flavonoid, oxalic acid, total antioxidant power, starch and soluble protein contents, as well as Ca and K levels, in heat-stressed wheat straw, concomitant with a decrease in lignin and phytic acid contents. In conclusion, the pronounced positive effects associated with improving yield quantity and quality were observed in stressed Si-treated wheat compared with Si nanoparticle-treated ones, particularly in cv. Gemmeiza 9.

## 1. Introduction

Food quality regularly evaluates the nutritional value and/or acceptability of a food product. Improving crop quality can help to improve the processing efficiency and nutritional value of crops. Wheat (*Triticum aestivum* L.) grain is an excellent staple food with nutritional and health beneficial compounds [1,2] that is composed of about 70% starch and 8–12 % protein contents, which are used for energy and growth [3]. Wheat grains can be ground into flour, which forms the essential constituents of bread and other bakery products [4]. The bread-making properties of flour are determined by dough properties, bread texture and elasticity, as well as water and oil absorptive capacities [5]. The water absorption capacity of flour represents the ability of a product to associate with water [6]. The oil absorption capacity of flour is helpful in mixing flour with oil as an essential ingredient [7].

Grain protein concentration and composition are also important quality factors that evaluate the nutritional value and, thereby, the efficiency of the bread-making process [8]. Meanwhile, the nutritional value is mostly determined by the synthesis of storage carbohydrates during grain filling. As temperatures rise above 18–22 °C, starch deposition is reduced, which ultimately reduces grain yield [9]. The highly important quality trait of flour is ∝-amylase level, which affects the bread-making properties of flour and is consistently added by the backing industry to improve dough properties [10], bread texture and elasticity [5,11].

One of the sustainable agricultural importance of wheat is increasing micronutrients and decreasing antinutrients, such as phytates, which inhibit micronutrient bioavailability and oxalic acid [12,13], the major chelator of calcium and inhibits its absorption in the human body [14,15]. Indeed, phytic acid (IP6) (phytate; inositol-hexaphosphate) is the primary source of inositol and storage phosphorus in plant seeds, which contributes ~70% of total phosphorus. Moreover, phytate has been reported as an antinutrient factor [16], antioxidant [17] and anticarcinogenic [18]. The protection of wheat plants from heat-induced oxidative damage during the reproductive phase has also been correlated with some antioxidants, such as flavonoids and phenols [19,20]. Wheat straw can be used as feedstock and for energy production, which could open new markets for improving the rural agriculture-based economy [21]. Furthermore, straw quality is a function of nutrient value [22].

Climate change represents a major challenge in the future that will intensively reduce crop yield [23] and the quality of staple grains [24]. Temperature flocculation during grain filling greatly affects the accumulation of protein in the developing wheat kernel and, consequently, alters the functional properties of the resulting flour, including dough properties and baking quality [13,25].

The sowing time of wheat is the most important factor for temperature-sensitive cereals such as wheat, barley, sorghum, etc. The optimum time for the sowing of wheat is mid-November until the first week of December; over 1 per cent yield loss per day takes place by delaying sowing after the first day of December [26]. Late sowing of wheat greatly reduces the yield as a result of low temperatures during germination and high-temperature stress during the reproductive stage [27]. Several studies have reported that high temperatures (>25 to 30 °C) during the grain-filling period significantly reduces grain yield, 1000-grain weight [28,29] as well as grain quality [30]. Consequently, searching for suitable and safe alleviators to meet the hazards caused by heat stress of late sowing is a very important issue, as well as improving the baking quality of wheat flour.

Silicon (Si) is one of the elements that has a valuable effect on plant growth and improves plant resistance to biotic stresses and abiotic stresses [31,32,33,34,35]. However, the involvement of Si nanoparticles (Si-Np) in the alleviation of heat stress is not yet well known. Recently, nanoparticles have been used for the improvement of individual life in several aspects, including agriculture, medicine, optics and mechanics [36,37,38,39,40]. Despite the information available on the nontoxicity of silicon nanoparticles [41] to plant systems, no studies have previously reported on improving the yield quality of plants grown under stress conditions.

Wheat gluten is the most important issue that determines the end product quality characteristics of wheat grain flour. Indeed, protein content and composition influence dough rheology and baking properties [42]. Gliadin and glutenin are the most important storage protein fractions of wheat grain. Glutenin subunits are about 30–45% of total wheat flour proteins and have a molecular weight ranging from 40 kDa to many millions. The wheat endosperm comprises high-molecular-weight (HMW) glutenin (80–140 kDa) and low-molecular-weight (LMW) glutenin (10–70 kDa) [43]. HMW glutenin subunits (HMW-GS) display the principal role in determining bread production quality, while LMW glutenin subunits (LMW-GS) are associated mainly with dough confrontation and flexibility [44]. High- and low-molecular-weight glutenin subunits can be linked via both inter- and intramolecular disulphide bonds, forming very large polymeric proteins [45].

Thus, the objective of the present study was to assess and compare the effects of Si-Np and Si on the mitigation of temperature stress induced by the late sowing of wheat plants and to explore their roles in improving the nutritional and desirable eating quality of wheat flour.

## 2. Results

### 2.1. Changes in Growth Parameters

The results presented in Table 1 and Table 2 show that heat stress induced by late sowing significantly reduced plant height, spike length, spike weight per plant, number of spikelets per spike, 1000-grain weight as well as grain yield per plant of both wheat cultivars. The application of either silicon or nanosilicon positively mitigated a reduction in previous growth parameters in both investigated cultivars (Table 1). However, such reductions were more pronounced in cv. Gemmeiza 9. Meanwhile, the harvest index was markedly decreased after exposure to the late sowing period in the two investigated cultivars (Table 2). Such a reduction was more pronounced in cv. Gemmeiza 9. The greatest increase in the harvest index was recorded in Si-treated wheat plants.

### 2.2. Changes in Nutritional and Antinutritional Values of Wheat Grains

Late sowing significantly increased the soluble sugar content in both investigated cultivars (Table 3). The percentage of increase was 47.7% in cv. Shandaweel 1 and 10.1% in cv. Gemmeiza 9. This increment was accompanied by a decrease in the contents of insoluble sugar and starch. The reduction in starch content was parallel with an increase in α-amylase activity (Table 3). The treatment with either silicon or nanosilicon increased the amount of soluble sugar, insoluble sugar as well as starch contents, concomitant with a significant reduction in α-amylase activity in both wheat cultivars exposed to heat stress induced by late sowing compared with normal sown ones. In addition, the levels of total soluble protein and insoluble protein in both investigated cultivars increased under heat stress (Table 4). Both silicon and nanosilicon induced a further increase in the contents of soluble and insoluble proteins in the grains of both cultivars.

**Figure 1:**
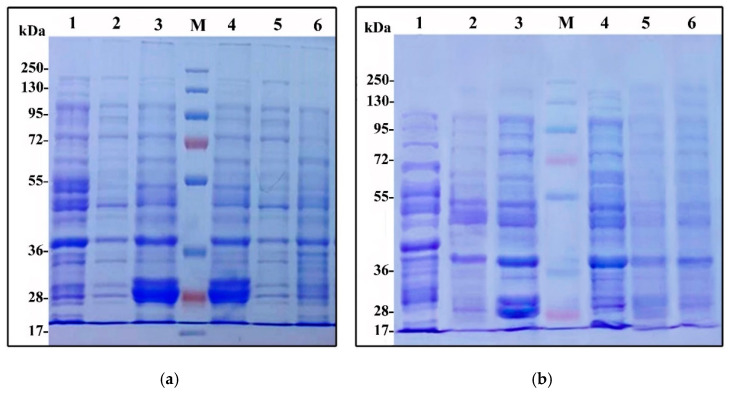
Electrograph SDS-PAGE of glutenin protein extracted from grains of two wheat cultivars treated with silicon and nanosilicon (**a**) cv. Shandaweel 1 and (**b**) cv. Gemmeiza 9: M, protein marker; Lane 1, control; Lane 2, 1.5 mM potassium silicate; Lane 3, 1.66 mM nanosilicon; Lane 4, heated 1.66 mM nanosilicon; Lane 5, heated 1.5 mM potassium silicate; Lane 6, heated control.

Additionally, the changes in glutenin content characterised by SDS gel electrophoresis are presented in Figure 1a,b. SDS-PAG analysis revealed the occurrence of some differences between the two investigated cultivars during the two manipulated dates. SDS-PAGE analyses of total glutenin polypeptides of Gemmeiza and Shandaweel wheat genotypes showed the presence of both high and low molecular glutenin subunits. The results also showed that cv. Gemmeiza 9 genotype comprises high glutenin subunits with a molecular weight of about 138 to 40 kDa in normal and late sowing wheat grain flour, respectively, compared with Shandaweel. Meanwhile, the Gemmeiza genotype exhibited a greater amount of high-molecular-weight glutenin subunits at the region of about 138 KDa compared with the Shandaweel genotype. On the other hand, the exposure of Gemmeiza and Shandaweel wheat genotypes to heat stress induced by late sowing stimulated marked reductions in the accumulation of glutenin subunits.

The foliar application of Si and NSPs reduced the accumulation of glutenin subunits in the two investigated wheat genotypes after exposure to heat stress induced by late sowing. However, a pronounced increment in the low-molecular-weight glutenin subunits (40–28 KDa) was detected in Si-applied grain flour, particularly during the normal growing season compared with the control and SiNPs.

**Figure 2:**
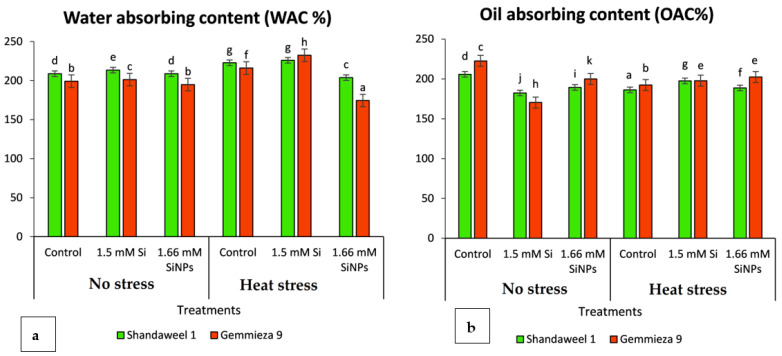
Effects of silicon and nanosilicon on (**a**) water absorption and (**b**) oil absorption contents of flour of two wheat cultivars cv. Shandaweel 1 and cv. Gemmeiza 9 at normal and late seasons. Each value is the main of three different replicates ± SE. Columns with different letters are significantly different at *p* < 0.05.

Data presented in Table 4 show that the levels of total phenols and flavonoids were significantly decreased under heat stress in the two investigated wheat cultivars. The total phenols were minimised in cv. Shandaweel 1 by 51.2%, while in cv. Gemmeiza 9 it was 1.80% compared with their corresponding controls. Similarly, the application of either silicon or nanosilicon reduced phenols and flavonoids in both heat-stressed wheat cultivars (Table 4). Additionally, heat stress induced by late sowing increased the WAC of both investigated wheat cultivars (Figure 2a). Meanwhile, Si treatment markedly enhanced the WAC of flour; however, nanosilicon reduced the same parameter, particularly in cv. Gemmeiza 9 exposed to late-season stressed conditions.

On the other hand, heat-induced stress during the late season significantly reduced the flour–oil absorbing capacity (OAC) of the two investigated wheat cultivars (Figure 2b). The application of Si and nanosilicon significantly increased the OAC of the flour of stressed wheat grains exposed to the late season compared with those of normal sowing plants.

Furthermore, the imposition of heat stress by late sowing significantly increased the concentrations of calcium (Ca), zinc (Zn) as well as phosphorous (P) levels in both the investigated cultivars compared with the controls. On the other hand, the potassium (K) level was decreased in both cultivars grown under heat stress conditions (Table 5).

The application of either silicon or nanosilicon displayed the same trend of Ca, Zn as well as P levels in both the investigated cultivars compared with their corresponding controls (Table 5). Meanwhile, the induced heat stress by late sowing brought a significant enhancement in inorganic P, concomitant with marked reductions in phytate P and phytic acid levels in Shandaweel 1 and Gemmeiza 9 grains (Table 6). In addition, foliar application with either silicon or nanosilicon had a positive influence on phytate P and phytic acid content in the grains of both stressed cultivars compared with those of untreated and treated grains in the normal season (Table 6).

### 2.3. Changes in Straw Yield and Quality

Heat stress induced by late sowing significantly decreased straw yield per plant in the two investigated wheat cultivars (Table 7). Such reduction was more pronounced in cv. Gemmeiza 9. However, Si and nanosilicon treatments markedly increased straw yield in late-sown plants.

Moreover, the insoluble sugar, insoluble protein contents as well as α-amylase activity declined in both late-sown wheat cultivars (Table 8). Such reductions were associated with increases in soluble sugar, starch as well as soluble protein contents in the two cultivars grown under stressed conditions induced by late sowing. The treatment with either silicon or nanosilicon decreased the levels of soluble sugar, insoluble sugar, insoluble protein and α-amylase activity compared with those of stressed wheat straw. However, the contents of starch and soluble protein were increased in the straw of the Si- and nanosilicon-treated stressed wheat cultivars.

**Figure 3:**
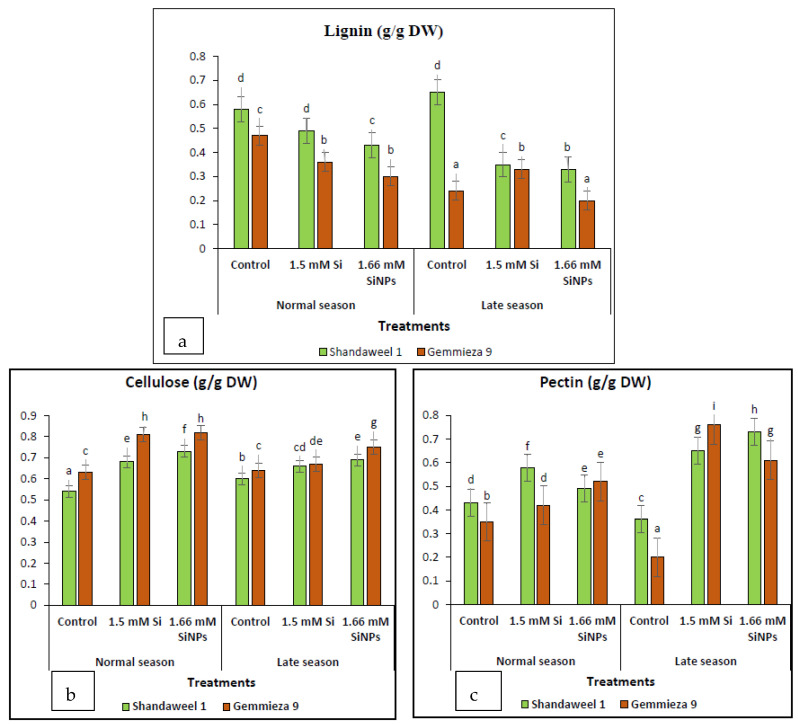
Effects of silicon and nanosilicon on (**a**) lignin, (**b**) cellulose and (**c**) pectin contents in straw of two wheat cultivars cv. Shandaweel 1 and cv. Gemmeiza 9 at normal and late seasons. Data expressed as (g/1g DW). Each value is the main of three different replicates ± SE. Columns with different letters are significantly different at *p* < 0.05.

Additionally, late sowing induced significant increases in total phenols, flavonoids, lignin and cellulose contents of the two investigated wheat straws (Table 8 and Figure 3a–c).

Such increments were accompanied by a decline in pectin and phytic acid contents (Figure 3c and Table 9).

Both silicon and nanosilicon provoked increments in total phenol, flavonoid, cellulose, pectin and phytic acid levels in heat-stressed wheat straw compared with those of untreated controls. However, lignin was markedly decreased in the treated plants compared with the corresponding controls (Figure 3a).

Late sowing-induced heat stress significantly reduced phosphorous (P) and zinc (Zn) levels in the two investigated straw wheat cultivars (Table 10). On the other hand, the levels of calcium (Ca) and potassium (K) were markedly increased in the two straw-stressed wheat cultivars. The foliar application of wheat grains in either silicon or nanosilicon had a positive influence on the accumulation of calcium and potassium levels in the two cultivars, particularly in late-sown wheat straw (Table 10). However, P and Zn were markedly decreased in Si- and nanosilicon-treated straw.

The level of oxalic acid accumulation in both wheat grains and straw was markedly decreased after exposure to the late heat-stressed season (Table 6 and Table 10). Meanwhile, Si and nanoSi treatments reduced the oxalic acid content in wheat grains, while it increased in straw.

The total antioxidant capacity was significantly increased in both wheat grains and straw exposed to heat stress induced by late sowing (Table 4 and Table 8). The application of either Si or nanosilicon significantly reduced total antioxidant capacity, particularly during the late season.

**Figure 4:**
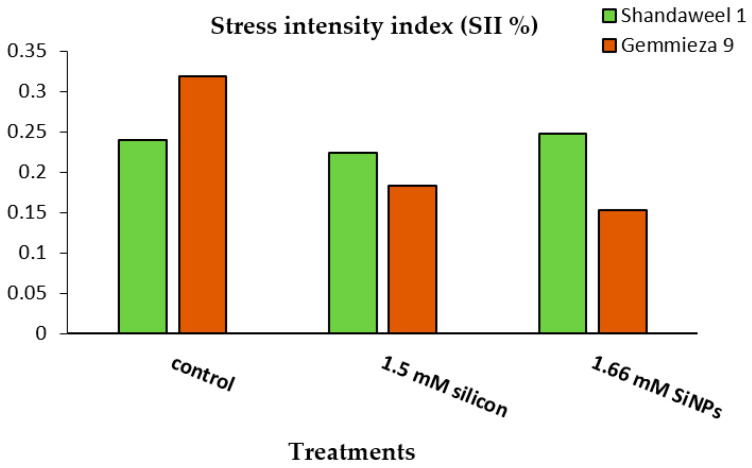
Effects of silicon and nanosilicon on stress intensity index (SII) of two wheat cultivars (Shandaweel 1 and Gemmeiza 9) grown under heat stress conditions.

Moreover, the stress intensity index was greater in cv. Gemmeiza 9 compared with cv. Shandaweel 1 (Figure 4). Foliar application with either Si or nanoSi markedly reduced the values of stress.

## 3. Discussion

The adverse environmental conditions during the anthesis and grain-filling period have been identified as major constraints to grain quality [46]. Sowing time is the most important factor for temperature-sensitive cereals [47] that causes a reduction in yield [48]. Late-sown wheat is affected at two stages: germination by low-temperature stress and at the reproductive stage by high temperature, which ultimately affects grain yield [49]. Few studies have evaluated the effects of heat stress induced by different sowing dates on the physicochemical qualities of wheat flour. Manipulation in sowing dates induces changes in the growth period, grain-filling duration and temperature, which is favourable for wheat growth. Thus, a normal sowing time has a significant effect on processing quality by controlling thermal conditions during growth and development [50]. As wheat is a major food crop in the world, the quality of the produced flour is a great challenge for the food industry. This has consequently increased the demand for the improvement of wheat flour, particularly wheat grown under stress conditions.

Planting wheat at its optimum sowing date would realise the optimum season length and achieve a high grain yield as a result of suitable weather conditions prevailing through different wheat growth stages [51]. Data of the present study revealed that sowing wheat in November is the optimum date for inducing the maximum yield traits at harvest in terms of spikelets/spike, grains number/spike, 1000-grain weight, grain yield and the harvest index (Table 1). These results are in good harmony with those obtained by [52,53,54]. However, late sowing showed a significant reduction in all yield components of wheat plants (Table 2). The reductions in these parameters might be attributed to the effect of the high temperature of late sowing at the reproductive stage, which leads to a shortening in the total growth duration, affecting grain maturity [55]. Similar results have been achieved by [56,57].

Late sowing reduced the number of grains per spike due to the unfavourable environment (temperature > 30 °C) at floret formation, which causes complete sterility and, ultimately, reduced grain filling [27,58,59]. Moreover, [29] reported a reduction in grain yield, 1000-grain weight and spikelet per spike as a result of high temperatures during the grain-filling period.

The reproductive phase of wheat seemed to be hampered by heat stress, which then affected the fertilisation process, leading to reduced grain yield (Table 2). This was probably due to the effect of grain-filling duration on dry matter accumulation [60]. In this respect, cereals generally respond to high temperatures through an increase in the rate of kernel growth, which leads to a decrease in the duration of dry matter accumulation [61,62]. Moreover, high temperatures affect endosperm development and reduce grain yield during endosperm cell division [63,64]. Additionally, [65] reported that high temperatures resulted in more immature grains and decreased yields in wheat by affecting both the source and sink for assimilates. A high harvest index is an indicator of the efficient utilisation of photosynthates [66,67]. Our results indicated that the percentage of the harvest index significantly decreased in grains of late sowing dates (Table 1). Such results might be attributed to decreases in tillers that develop during spring and are small with a low harvest index [68]. The authors of [69] reported that there is a positive and significant correlation between the stress intensity index (SII) and the productivity index. A higher SII value is an indicator of lower tolerance to stress [70]. Based on this index, stressed cv. Gemmeiza 9 was less tolerant compared with cv. Shandaweel 1 with respect to heat stress tolerance. Meanwhile, SII was markedly reduced in stressed Si- and nanosilicon-applied wheat plants. Both Si and nanosilicon foliar applications markedly nullified the observed decline in yield components at late sowing dates (Table 1). Such a finding is confirmed by [71], who investigated that under this condition, leaves become more erect, thus reducing self-shading and increasing the rate of photosynthesis, which enhances sink activity and better dry partitioning, thereby enhancing yield components, particularly during the period of grain filling that is associated with starch accumulation in grains. Moreover, increasing grain yield in Si-treated wheat plants under heat stress conditions might be attributed to the increase in the biomass accumulation [72,73] and/or stimulation of 1000-grain weight [74].

Proteins are the most important components of wheat grains, governing end-use quality [75]. Although grain protein composition depends primarily on the genotype, it is also significantly affected by environmental factors [76]. The results shown in Table 4 indicated that higher soluble protein contents were recorded in late-sown yielded wheat grains, which might be due to the increase in protein synthesis and decrease in its degradation [77]. Our results were in accordance with [78]. Moreover, [79] also found that barley grain protein content significantly increased by delaying the sowing date. Variations in protein content significantly modify grain quality [80]. In fact, an increase in grain protein content improves the nutritional value; however, it gives a very bad taste [81]. In addition, [82] suggested the possible role of proteins as potential chelators for some micronutrients. Therefore, an increase in total soluble protein content in wheat grains is considered a step in improving nutritional grain quality, and this is the case in Si- and nanosilicon-treated plants. Our results were in accordance with [74], who reported that the application of Si improves soluble protein content in rice through the translocation of different nutrients such as N, which enhances nitrogen metabolism and the synthesis of proteins in plants. Moreover, the accumulation of protein in response to silicon may be attributed to the role of Si in specific protein synthesis, functioning of mRNA and DNA synthesis [83]. Moreover, Si provides a dehydration tolerance to plant cells and accelerates the assimilation of photosynthates [31]

Increasing temperatures during grain filling increase the protein content and dough strength [84,85]; however, high temperatures (30 °C) alter the composition of both protein and starch within the grain. Similarly, [86] demonstrated that carbohydrate metabolism was shifted towards protein accumulation under a shorter duration of grain development associated with high temperatures [87]. Meanwhile, both silicon and nanosilicon increased total soluble and insoluble proteins in grains of both cultivars (Table 2), which increased the nutritional value and dough strength.

Glutenin is composed of high-molecular-weight glutenin subunits (HMW-GS) and low-molecular-weight glutenin subunits (LMW-GS), both of which affect the strength and elasticity of wheat dough [43,88]. Notably, the SDS-PAG analysis revealed the occurrence of marked differences between the two investigated genotypes during the two manipulated dates. The two wheat genotypes showed reductions in the glutenin subunits during the late sowing date, which may play a role in determining the quality characteristics of wheat grain flour, as determined by [89], who postulated the positive relation between glutenin subunits, particularly low-molecular-weight glutenin, and the good quality of bread wheat. The detected variation in electrophoretic bands of glutenin subunits of the investigated genotypes may be due to the differences in their genetic composition [90]. Such results are in harmony with those of [90,91,92], who reported that low molecular glutenin subunits (32.3 to 67.4 kDa) represented about 60% of the total wheat glutenin fractions of different wheat genotypes. It was reported that gluten is a determinant of the elasticity and plasticity of most flour-based products [93]. Heat-induced treatment induced by the late sowing of wheat grains alters the protein and starch contents, so rheological characteristics and pasting properties are also changed [94,95]. The current findings are in conformity with those reached by [93], who indicated that environmental conditions affect the amount, composition and/or polymerisation of gluten proteins [96]. Similarly, the electrophoretic characteristics of gluten proteins are affected by crop year and variety [90,97].

Moreover, the application of Si treatments persuaded the synthesis of low-molecular-weight glutenin subunits with an MW of about 32 kDa in both investigated genotype grains exposed to normal or heat stress induced by late sowing. Such increments in the concentration and types of low-molecular-weight glutenin subunits induced by SI and nanoSi application induced by late sowing may have good physical dough properties of flour during bread-making, as reported by [90,98].

Fluctuation in temperatures by the manipulation of sowing dates resulted in increments in the soluble carbohydrates of wheat grains sown at late dates (Table 4). Such an effect might probably be driven by the different thermal conditions prevailing during the grain filling period. Increments in the sugar content of wheat grains may also be due to its mobilisation from the stem, which is enhanced at high temperatures during wheat grain filling [75,99,100]. Additionally, increments in soluble carbohydrates were concomitant with reductions in the starch content of the yielded wheat grains at late sowing dates (Table 4). The reduction in starch accumulation might have resulted from the limited supply of assimilates for the grain or loss of activity of starch synthesis-related enzymes [87,101,102,103]. On the other hand, the Si and nanosilicon enhancement effect was attributed to its effect on the stimulation of chlorophyll formation and the protection of photosynthetic apparatus [73]. Moreover, Si plays an important role in regulating transpiration and the uptake of some micro- and macronutrients [104]. In addition, Si has been reported to exert influence on partitioning photosynthates to the sites of flowering and fruit production [105]. It has been reported that silicon decreases oxidative destruction caused by abiotic stresses, as it has the ability to scavenge reactive oxygen species (ROS) by improving antioxidant enzymatic activities and the accumulation of some osmolytes, such as soluble sugars [106], and consequently decrease the damage caused by heat stress.

Data in the present investigation showed that the greatest α-amylase activity was achieved in the late season (Table 4). The increment in α-amylase activity in stressed wheat grains was concomitant with starch reduction. Data also showed that Si sprayed significantly decreased the activity of α-amylase. It is also evident from the result that varieties that showed higher α-amylase activity under late sowing had lower starch content and vice versa. Consequently, poor bread quality of wheat is automatically related to low yield or high α-amylase levels [107]. Several studies have demonstrated the beneficial effect of α-amylase on bread texture and elasticity [5,11] by generating greater quantities of fermentable sugar [108]. Heat stress induced by late sowing increased the WAC of both investigated wheat cultivars. Meanwhile, Si treatment markedly enhanced the WAC of flour. Such an increase leads to the production of more moist and soft textured bread. However, nano Si treatment reduced the same parameter, particularly in cv. Gemmeiza 9 exposed to late-season heat-stressed conditions.

Water absorption capacity (WAC) measures the ability of flour to absorb water and swell for improved stability in food. Water absorption capacity is important in bulking and consistency of products as well as in baking applications [109]. Late sowing significantly increased the soluble sugar content in both investigated cultivars, particularly in cv. Gemmeiza 9, which enhanced the water absorption capacities of flours since these constituents contain hydrophilic parts, such as charged side chains that have an affinity for water molecules.

Heat-induced stress during the late season significantly reduced the flour–oil absorption capacity (OAC) of the two investigated wheat cultivars (Figure 4). In contrast, the application of Si and nanosilicon significantly increased the OAC of the flour of stressed wheat grains exposed to the late season compared with those of normal sowing plants. Oil absorption capacity (OAC), on the other hand, is important because it acts as a flavour retainer. A higher oil absorption capacity suggests the lipophilic nature of flour constituents [110]. The major chemical component affecting OAC is protein, which is composed of both hydrophilic and hydrophobic parts. In this context, Si and nanosilicon stimulated a further increase in soluble and soluble proteins (Table 4). Thus, a larger proportion of hydrophilic groups or polar amino acids on the surface of protein molecules tend to cause an increase in the oil absorption capacity of flours since the protein in food affects fat absorption [111].

Additionally, the amount of different minerals in the grain depends on the levels transported by roots during grain development [112]. Data presented in this investigation revealed that the levels of Ca^2+^ content increased in the yielded grains of late-sown wheat (Table 6). This may be attributed to the influence of heat stress induced by late sowing on Ca^2+^ mobilisation during grain filling. In this respect, [113] reported that Ca^2+^ assimilation to the shoots and spikes increased in heat-stressed wheat, alleviating heat injury [114] and limiting oxidative damage [115].

The high negative charge density of phytic acid acts as a strong chelator of positively charged mineral cations such as Zn^+2^ [116]. Thereby, Zn^+2^ content increased in late-sown grains, concomitant with reductions in phytic acid content (Table 6). Similar results have been obtained by [13]. Food crop breeding strategies for higher levels of nutrients and low levels of antinutritional substances, such as phytic acid and oxalic acid, have been reported [12]. It is clearly obvious that heat conditions resulted not only in low phytate P and phytic acid but also in high grain inorganic P (Pi) contents. This result probably could be due to the fact that phytic acid has been viewed primarily as a phosphate storage compound [117]. The observed increase in the grain Pi of heat-stressed plants was concomitant with a decrease in starch and, consequently, grain yield (Table 2 and Table 3). Such an effect might be attributed to the inhibition of starch synthesis by high inorganic phosphate (Pi) [118]. Under stress conditions, IP3 accumulates [119], resulting in the release of Ca^2+^ from intercellular stores and, consequently, stomatal closure [120]. The heat stress-induced pathway in the two investigated cultivars does not involve the phosphorylation of IP3; therefore, the observed decrease in IP6 (phytate) was parallel with the observed increase in Ca^2+^ [121]. It could be concluded that phytate has no direct relationship with the tolerance of wheat. Both Si and nanosilicon significantly caused further accumulation of Ca ions in stressed wheat. It is concluded that Si has a beneficial effect on the accumulation of macronutrients such as Ca^2+^. In fact, Si increases Ca^2+^ uptake [122]. Potassium decreased in late-sown wheat grains in both investigated cultivars (Table 8). Such an effect might be attributed to heat stress-induced alterations in K ion mobility, and/or the allocation of this nutrient may be shifted to another part in heat-stressed plants [123]. Meanwhile, Si and Si nanoparticles markedly decreased compared with their corresponding controls. Silicon plays an important role in dissociating phytate–protein/mineral complexes, which are involved in the reduction in antinutritional quality under environmental stress [124]. Moreover, nanoSi improved the absorption and translocation of nutrients such as Ca^2+^ in response to abiotic stress; this might be attributed to mineral uptake depending on membrane activity, which plays an important role in the transport of nutrients from the soil to the plant, enabling an improvement in the physiological and biochemical activities of the plant [125].

Plant phenols and flavonoids are multifunctional and can act as reducing agents, hydrogen donors and singlet oxygen quenchers [126]. The data revealed that late sowing decreased the accumulation of both phenols and flavonoids in wheat grains (Table 4). The results also showed that the antioxidant capacity of yielded grains increased in late-sown plants (Table 4). Our results are in good harmony with the findings of [127,128]. It could be concluded that both phenols and flavonoids have no direct relationship with the observed increase in TAC of wheat grains.

Grain yield was strongly related to straw production, and both were positively related to plant height (Table 2). It is of interest to mention here that the observed decrease in straw yield (g) per plant under heat stress was accompanied by a high percentage of lignin and cellulose (Table 8 and Figure 3a,b). Heat stress induced by late sowing accelerated lignification and cellulose accumulation in wheat straw. Si and nanosilicon treatments caused an increase in cellulose, lignin and pectin contents compared with stressed plants. This might be attributed to the improvement of carbohydrate production by silicon treatment [129]. The chemical composition of wheat straw varied significantly with the variety and stress conditions [130,131,132].

The results also revealed that the late sowing date increased total soluble protein contents in wheat straw, which might be attributed to heat stress, which contributed to maintaining cell structure and function under stress conditions.

In addition, changing the sowing date influenced the mineral contents of wheat straw (Table 10). The level of Ca^2+^ content significantly increased in late-sown wheat straw as a mechanism of heat tolerance in wheat, which is associated with the maintenance of net carbon assimilation rates [113]. On the other hand, the K^+^ level increased in late-sown wheat straw (Table 10). The reduction in K^+^ levels in grains was accompanied by an increase in its level in straw (Table 6 and Table 10).

The data indicated that straw yield had a negative relationship with antioxidant capacity. It is well known that heat stress, similar to other stresses, induces increments in the production of reactive oxygen species (ROS), which, in excess, could be harmful to plant cells. Natural plant antioxidants, such as phenolics, are involved in the regulation of ROS content. According to data from the early literature, the antioxidant capacity of straw is highly positively related to phenolic and flavonoid contents [133].

In conclusion, the responses of the two investigated wheat cultivars to late sowing were different cv. Shandaweel1 showed good cooking and eating characteristics as well as high yield potential under induced heat stress. However, the mechanism of grain quality improvement needs further elucidation, particularly on the molecular level. Si and nanoSi mitigated the adverse effect of temperature, improved the nutritional quality of grain and straw as well as maintained favourable agronomic quality.

## 4. Materials and Methods

### 4.1. Plant Material and Growth Conditions

The experiment was conducted at the botanical garden, Botany Department, Faculty of Science, Ain Shams University, Cairo, Egypt. The experiment was carried out between December 2017 and February 2018. Two local wheat (*Triticum aestivum* L.) cultivars were utilised in this study, namely, Shandaweel 1 (heat-tolerant) and Gemmeiza 9 (heat-sensitive) genotypes. The cultivation area used was divided into two plots, each of which was divided into three subplots; each subplot was 1 m^2^ in area. Wheat plants were foliar sprayed with potassium silicate and silicon nanoparticles at a concentration of 1.5 mM and 1.66 mM, respectively, after 50 and 70 days from sowing. Fluctuated temperature stress was induced by manipulating the date of sowing. The grains were sown on two different dates: 26 December (normal date) and 4 February (late date). The experiment was conducted in a randomised complete block design (RCBD) with a split-plot arrangement and three replications. At full maturity (120 and 90 days for normal and late-sown wheat, respectively), the experiment was terminated, and 10 randomised plants were harvested from each treatment for carrying out the following measurements: number of grains/spike, 1000-grain weight (g), biological yield (g/plant), grain yield (g/plant) and harvest index (%). Moreover, grain and straw qualities were investigated.

### 4.2. Methods

#### 4.2.1. Stress Intensity Index (SII)

The stress intensity index was calculated using the formula in [134].

SII=1−( grain yield in stress condition/grain yield in control condition)

#### 4.2.2. Harvest Index (HI)

HI = Economic yield (grain yield)/Biological yield (aboveground dry matter) × 100 [135]

#### 4.2.3. Water Absorption Capacity

The water absorption capacity (WAC) was determined according to a combination of [136] and those of [137,138]. Two grams of grains were taken in graduated test tubes with 10 mL of water and soaked for 30 min, then boiled for 45 min at 77 to 80 °C in a constant temperature water bath and cooled in a beaker containing cold water. The supernatant was poured into a graduated cylinder after cooling, and the water level was noted. Water absorption capacity was calculated using the following formula:

Water absorption capacity= 100/2 g × actual water absorbed

#### 4.2.4. Oil Absorption Capacity

The oil absorption capacity (OAC) was determined by the method adopted by [139]. Four grams of flour sample was mixed with 20 mL peanut or canola oil in a centrifuge tube and stirred with a glass rod for 30 min at 24 °C, then centrifuged at 15.000*g* for 15 min. The supernatant was measured as millilitres of oil per gram of flour sample used.

#### 4.2.5. Determination of Carbohydrates

The soluble carbohydrate and starch contents were determined with an anthrone reagent according to the method adopted by [140]. A known volume of plant extract (2 mL) was mixed with 10 mL of anthrone reagent and then boiled in a water bath for 15 min; after cooling, the absorbance was read at 620 nm by spectrophotometer. Moreover, the method used for the determination of insoluble carbohydrate was determined as described by [141]. A known weight of dried sample was transferred to sugar tubes with 1.5 N H_2_SO_4_ for 6 h at 100 °C. The hydroslate solution was used for insoluble sugar by anthrone reagent. A calibration curve was conducted by glucose.

#### 4.2.6. Assay of α-Amylase Activity

The α-amylase activity was assayed by the method described by [142]. Soluble starch as a substrate. 100 μL of plant enzyme extract was added to 20 μL of 1% starch solution, then the mixture was incubated at 37 °C for 1 h, and then 1% iodine solution (20 μL) was added; finally, the absorbance was read at 565 nm by spectrophotometer.

#### 4.2.7. Determination of Nitrogenous Constituents

Total soluble protein and insoluble protein were measured by using Folin–Ciocalteu reagent, based on the method described by [143]. Five mL of alkaline reagent was mixed with 1 mL of soluble protein extract and allowed to stand for 10 min at room temperature. Then, 0.5 mL of Folin–Ciocalteu reagent was added, and the absorbance was read at 700 nm after 30 min. Moreover, the water-insoluble residue remaining after extraction of soluble sugar was digested with 1 N NaOH for 20 min. The insoluble protein extract was determined by Folin–Ciocalteu reagent, as mentioned before. A calibration curve was constructed by egg albumin.

#### 4.2.8. Determination of Flavonoids

The method used for the determination of flavonoids was that of [144]. A known volume of plant extract (1 mL) was added to 0.1 mL of potassium acetate, 1.5 mL methanol, 0.1 mL 10% aluminium chloride and 2.8 mL distilled water and then incubated for 30 min at room temperature. The absorbance was measured at 415 nm by spectrophotometer. The flavonoid content was calculated from a standard curve of quercetin.

#### 4.2.9. Determination of Total Phenols

The method applied for the determination of total phenols was that of [145]. A known volume of phenol extract (0.5 mL) was added to 0.5 mL of Folin–Ciocalteu reagent (for phenol). The mixture was allowed to stand for 3 min, and then 1 mL of sodium carbonate (25%) was added; then, it was further allowed to stand for 60 min at room temperature. Finally, the absorbance was measured at 725 nm by spectrophotometer. The number of total phenols was calculated according to the gallic acid standard curve.

#### 4.2.10. Determination of Total Antioxidant Capacity (TAC)

Total antioxidant capacity was determined according to the method described by [146]. One mL of plant extract was added to 1 mL of 0.2M sodium phosphate buffer (pH 6.6) and 1 mL of 1% potassium ferricyanide; the mixture was incubated in a water bath at 50 °C for 20 min, followed by the addition of 1 mL of 10% trichloroacetic acid. Centrifugation of the mixture was carried out at 5000× *g* for 10 min, after which 1 mL was taken from the supernatant and mixed with 1 mL of deionised water and 200 μL of 0.1% FeCl_3_. The absorbance was measured at 700 nm by spectrophotometer.

#### 4.2.11. Determination of Certain Minerals

Minerals were extracted following the method of [147]. A total of 2 g of dried plant tissue was placed in a digestion flask under a ventilated hood, and then 20 mL of a triple acid mixture (750 mL of nitric acid, 150 mL of sulfuric acid and 300 mL of perchloric acid) was added and then heated on a hot plate at low temperature for several minutes; then, the temperature was increased until fumes of sulfuric acid evolved. Calcium, potassium, phosphorous and zinc levels were estimated by using an atomic absorption spectrophotometer according to the method of the [148].

#### 4.2.12. Determination of Phytate Phosphorous and Phytic Acid Contents

Phytate phosphorous was extracted and estimated according to the method described by [149]. In total, 10 mL of extract was added to 4 mL of FeCl_3_ solution, and then the mixture was heated in a boiling water bath for 45 min. After cooling, 5 mL was transferred to another volumetric flask and diluted to 75 mL and added 20 mL of 1.5M potassium thiocyanate. The absorbance was read at 480 by spectrophotometer within 1 min.

Phytate P was calculated from the standard curve of Fe(NO_3_)_3_ according to the following equation:

mg/100 g sample = µg Fe × 15/ weight of sample (g)

Phytic acid was then calculated using the factor 3.55 [150].

#### 4.2.13. Determination of Oxalate

Oxalate was estimated according to the titration method implemented by [151]. One gram of plant tissue was added to 75 mL of 3 M H_2_SO_4_ and stirred for 1 h with a magnetic stirrer. After filtration, 25 mL of filtrate was titrated against 0.05 M KMNO_4_ solution until a faint pink colour. The oxalate content was then calculated by taking 1 mL of 0.05 M KMnO_4_ as equivalent to 2.2 mg oxalate [152].

#### 4.2.14. Determination of Lignin, Cellulose and Pectin

The straw quality of lignin, cellulose and pectin were determined by the methods of [109,153,154], respectively. Lignin was extracted by weighing 3 g of dried plant tissue and extracted with a benzene-alcohol mixture (67:33 *v*/*v*) for 6 hrs. After drying, the extract fibres were digested with cold 72% H_2_SO_4_ for 3.5 hrs at room temperature. The acid mixture was diluted with water and then filtered. The residue was dried and weighed. The resultant residue formed the lignin content of the sample. Moreover, the cellulose was determined by immersing 3 g of dried sample with 1 % sodium hydroxide solution and boiling in a water bath for 5 min. After filtration, the residue was treated with 1% HCl, then with 5 mL of sodium hypochlorite (15%), then with sodium hydroxide and kept in the dark for 20 min, and then washed with water then hydrogen peroxide. Finally, the resultant residue was dried at 105 °C to constant weight. On the other hand, the pectin was extracted by mixing 3 g of sample with 200 mL of 0.5 % ammonium oxalate solution for 24 h at 85 °C. The mixture was filtered, and then a known volume (100 mL) of the filtrate was added to 3.5 mL of 95% ethanol and six drops of conc. HCl. After 12 h, the precipitate was filtered and then added to 50 mL of 1 N acetic acid and 50 mL of calcium chloride solution. The calcium pectate was filtered, and the precipitate was dried until constant weight.

#### 4.2.15. Glutenin Profile

The glutenin protein was extracted according to the method of [155]. In total, 0.1 g of wheat flour was extracted in 0.4 mL of KCl buffer (pH 7.8) to eliminate the soluble proteins. The insoluble protein–KCl fraction was suspended in 1-propanol solution (50% *v*/*v*) and centrifuged at 4500× *g* for 10 min to eliminate gliadins from glutenins. The composition of glutenin components (10 μL) was separated by acid polyacrylamide gel electrophoresis (SDS-PAGE). Relative mobility and colouring intensity of the components of glutenin were estimated by the protein by gel documentation system (Syngene, Cambridge, UK). On the first well of each gel, the proteins were employed as the molecular weight (Daltons) markers ranging from 17–250 KDa.

### 4.3. Statistical Analysis

Statistical analysis was performed using a one-way analysis of variance (ANOVA). The data were subjected to a least significant difference test (LSD) according to [156]. Then Duncan’s test was applied at a 0.05 probability level to compare between groups.

## 5. Conclusions

In conclusion, the responses of the two investigated wheat cultivars to late sowing were different. The cv. Shandaweel 1 showed good cooking and eating characteristics as well as high yield potential under induced heat stress. However, the mechanism of grain quality improvement needs further elucidation, particularly on the molecular level. Si and nanoSi mitigated the adverse effect of temperature, improved the nutritional quality of grain and straw as well as maintained favourable agronomic quality. Good bread quality of wheat is related to high yield and low α-amylase activity in Si- or nanoSi-treated heat-stressed plants, which is considered a step in improving grain quality. Si application markedly enhanced the WAC of flour. Such an increase leads to the production of more moist and soft textured bread. On the other hand, Si and nanoSi significantly increased the OAC of the flour of stressed wheat grains exposed to the late season, which is important because it acts as a flavour retainer. It is also concluded that phytate, phenols and flavonoids have no direct relationship with the tolerance of wheat and the concomitant observed increase in TAC of wheat grains.

## Figures and Tables

**Table 1:** Effects of silicon and nanosilicon on yield components (spike length, spike weight, number of spikelets per spike) of two wheat cv. Shandaweel 1 and cv. Gemmeiza 9 at normal and late seasons. Each value is the main of 10 different replicates ± SE.

Treatments			Spike Length (cm)	Spike Weight (g)	Number of Spikelets/Spike
Normal season	Shandaweel 1	Control	8.8 ± 0.12 ^g^	0.66 ± 0.005 ^i^	28.0 ± 0.33 ^d^
		1.5 mM silicon	10.8 ± 0.14 ^j^	1.76 ± 0.006 ^f^	36.0 ± 0.33 ^g^
		1.66 mM nanosilicon	10.0 ± 0.03 ^i^	1.57 ± 0.01 ^e^	35.0 ± 0.33 ^g^
	Gemmeiza 9	Control	7.5 ± 0.05 ^e^	0.54 ± 0.005 ^c^	28.0 ± 0.001 ^d^
		1.5 mM silicon	9.2 ± 0.06 ^h^	2.07 ± 0.02 ^h^	38.0 ± 0.33 ^h^
		1.66 mM nanosilicon	8.8 ± 0.08 ^g^	1.82 ± 0.03 ^g^	30.0 ± 0.33 ^e^
Late season	Shandaweel 1	Control	6.5 ± 0.05 ^d^	0.43 ± 0.02 ^b^	26.0 ± 0.33 ^c^
		1.5 mM silicon	7.8 ± 0.03 ^f^	0.71 ± 0.018 ^d^	32.0 ± 0.33 ^f^
		1.66 mM nanosilicon	6.9 ± 0.15 ^d^	0.57 ± 0.005 ^c^	28.0 ± 0.33 ^d^
	Gemmeiza 9	Control	4.7 ± 0.08 ^a^	0.28 ± 0.008 ^a^	22.0 ± 0.33 ^a^
		1.5 mM silicon	5.9 ± 0.03 ^c^	0.45 ± 0.012 ^b^	28.0 ± 0.33 ^d^
		1.66 mM nanosilicon	5.3 ± 0.12 ^b^	0.38 ± 0.012 ^a^	24.0 ± 0.66 ^b^

Values with different letters in column are significantly different at *p* < 0.05.

**Table 2:** Effects of silicon and nanosilicon on yield components (Weight of 1000-grains, Grain yield/p, HI) of two wheat cv. Shandaweel 1 and cv. Gemmeiza 9 at normal and late seasons. Each value is the main of 10 different replicates ± SE.

Treatments			Weight of 1000-Grains	Grain Yield/p	HI (%)
Normal season	Shandaweel 1	Control	30.3 ± 0.06 ^g^	32.1 ± 0.12 ^e^	94.1 ± 0.12 ^k^
		1.5 mM silicon	37.2 ± 0.08 ^j^	40.1 ± 0.145 ^g^	96.3 ± 0.24 ^l^
		1.66 mM nanosilicon	35.4 ± 0.03 ^i^	39.8 ± 0.057 ^g^	99.7 ± 0.20 ^e^
	Gemmeiza 9	Control	29.6 ± 0.08 ^f^	24.6 ± 0.26 ^c^	89.1 ± 0.06 ^c^
		1.5 mM silicon	42.1 ± 0.18 ^l^	39.0 ± 0.152 ^h^	98.4 ± 0.14 ^g^
		1.66 mM nanosilicon	38.2 ± 0.057 ^k^	33.9 ± 0.272 ^f^	92.8 ± 0.94 ^a^
Late season	Shandaweel 1	Control	22.9 ± 0.152 ^d^	24.4 ± 0.133 ^b^	88.0 ± 0.26 ^h^
		1.5 mM silicon	32.1 ± 0.08 ^h^	31.1 ± 0.484 ^e^	95.3 ± 0.26 ^i^
		1.66 mM nanosilicon	26.0 ± 0.03 ^e^	28.5 ± 0.348 ^d^	92.8 ± 0.47 ^b^
	Gemmeiza 9	Control	14.3 ± 0.115 ^a^	16.5 ± 0.088 ^a^	83.7 ± 1.39 ^f^
		1.5 mM silicon	21.8 ± 0.185 ^c^	31.3 ± 0.47 ^f^	97.2 ± 0.43 ^j^
		1.66 mM nanosilicon	17.7 ± 0.145 ^b^	27.8 ± 0.328 ^c^	95.2 ± 0.21 ^d^

Values with different letters in column are significantly different at *p* < 0.05.

**Table 3:** Effects of silicon and nanosilicon on soluble, insoluble sugar, starch and α-amylase activity (mg sugar/min./g DW) of two wheat cultivars grains cv. Shandaweel 1 and cv. Gemmeiza 9 at normal and late seasons. Each value is the main of three different replicates ± SE.

Treatments			Soluble Sugar	Insoluble Sugar	Starch	α-Amylase
Normal season	Shandaweel 1	Control	4.98 ± 0.052 ^c^	23.31 ± 0.057 ^b^	18.3 ± 0.088 ^a^	50.4 ± 0.218 ^k^
		1.5 mM silicon	7.64 ± 0.020 ^g^	31.86 ± 0.061 ^d^	21.0 ± 0.057 ^b^	43.6 ± 0.088 ^h^
		1.66 mM nanosilicon	6.65 ± 0.023 ^f^	28.79 ± 0.065 ^c,d^	20.4 ± 0.116 ^c^	47.1 ± 0.218 ^g^
	Gemmeiza 9	Control	4.82 ± 0.012 ^c^	25.8 ± 0.057 ^b,c^	14.4 ± 0.100 ^a^	48.0 ± 0.240 ^l^
		1.5 mM silicon	5.30 ± 0.038 ^d^	30.75 ± 0.125 ^d^	19.0 ± 0.218 ^b^	36.9 ± 0.208 ^j^
		1.66 mM nanosilicon	5.43 ± 0.028 ^d^	35.66 ± 0.041 ^e^	18.3 ± 0.185 ^d^	35.1 ± 0.088 ^i^
Late season	Shandaweel 1	Control	7.36 ± 0.211 ^g^	15.65 ± 0.025 ^a^	10.1 ± 0.266 ^d^	67.1 ± 0.264 ^c^
		1.5 mM silicon	8.51 ± 0.020 ^e^	19.44 ± 1.190 ^a^	16.3 ± 0.1 ^h^	45.9 ± 0.202 ^h^
		1.66 mM nanosilicon	9.05 ± 0.008 ^e^	18.73 ± 0.312 ^a^	14.5 ± 0.115 ^g^	49.0 ± 0.133 ^e^
	Gemmeiza 9	Control	5.31 ± 0.006 ^d^	18.96 ± 0.111 ^a^	10.6 ± 0.185 ^e^	69.6 ± 0.066 ^d^
		1.5 mM silicon	6.73 ± 0.012 ^a^	28.86 ± 3.290 ^b,c^	18.4 ± 0.173 ^f^	37.2 ± 0.218 ^b^
		1.66 mM nanosilicon	6.42 ± 0.038 ^b^	32.73 ± 1.455 ^d^	17.9 ± 0.360 ^i^	39.7 ± 0.033 ^a^

Values with different letters in column are significantly different at *p* < 0.05.

**Table 4:** Effects of silicon and nanosilicon on soluble protein, insoluble protein total phenol, flavonoid and total antioxidant capacity (TAC, µg/g DW) of two wheat cultivars grains cv. Shandaweel 1 and cv. Gemmeiza 9 at normal and late seasons. Each value is the main of three different replicates ± SE.

Treatments			Soluble Protein	Insoluble Protein	Total Phenols	Flavonoids	Total Antioxidant Capacity
Normal season	Shandaweel 1	Control	1.45 ± 0.014 ^g^	2.75 ± 0.017 ^b^	1.93 ± 0.018 ^g^	0.49 ± 0.015 ^f^	0.23 ± 0.036 ^a^
		1.5 mM silicon	2.25 ± 0.043 ^k^	3.0 ± 0.003 ^c,d^	1.44 ± 0.017 ^f^	0.31 ± 0.008 ^d^	0.38 ± 0.005 ^c^
		1.66 mM nanosilicon	2.79 ± 0.012 ^j^	3.37 ± 0.012 ^e^	1.32 ± 0.006 ^e^	0.28 ± 0.006 ^c^	0.32 ± 0.006 ^b^
	Gemmeiza 9	Control	1.33 ± 0.013 ^a^	2.58 ± 0.012 ^a^	1.11 ± 0.012 ^d^	1.03 ± 0.014 ^g^	0.27 ± 0.001 ^a^
		1.5 mM silicon	1.71 ± 0.014 ^d^	2.71 ± 0.017 ^b^	0.87 ± 0.003 ^b^	0.44 ± 0.007 ^e^	0.31 ± 0.016 ^b^
		1.66 mM nanosilicon	2.00 ± 0.013 ^e^	3.0 ± 0.040 ^d^	0.82 ± 0.016 ^b^	0.25 ± 0.006 ^b,c^	0.41 ± 0.001 ^d^
Late season	Shandaweel 1	Control	2.42 ± 0.003 ^b^	2.97 ± 0.037 ^c^	0.94 ± 0.012 ^c^	0.31 ± 0.006 ^d^	0.52 ± 0.001 ^f^
		1.5 mM silicon	10.0 ± 0.005 ^f^	3.95 ± 0.035 ^h^	0.75 ± 0.003 ^a^	0.21 ± 0.013 ^b^	0.48 ± 0.008 ^e^
		1.66 mM nanosilicon	5.87 ± 0.008 ^h^	3.67 ± 0.008 ^g^	0.86 ± 0.006 ^b^	0.24 ± 0.005 ^b,c^	0.38 ± 0.002 ^c,d^
	Gemmeiza 9	Control	1.66 ± 0.003 ^c^	3.33 ± 0.005 ^e^	1.09 ± 0.010 ^d^	0.48 ± 0.018 ^e,f^	0.60 ± 0.005 ^g^
		1.5 mM silicon	2.71 ± 0.026 ^h^	3.58 ± 0.008 ^f^	0.76 ± 0.078 ^b^	0.28 ± 0.012 ^c^	0.35 ± 0.003 ^bc^
		1.66 mM nanosilicon	3.12 ± 0.025 ^i^	3.95 ± 0.011 ^h^	0.80 ± 0.032 ^a,b^	0.12 ± 5.62 ^a^	0.44 ± 0.001 ^e^

Values with different letters in column are significantly different at *p* < 0.05.

**Table 5:** Effects of silicon and nanosilicon on calcium (Ca), zinc (Zn), potassium (K) and phosphorous (P) in grains of two wheat cv. Shandaweel 1 and cv. Gemmeiza 9 at normal and late seasons. Each value is the main of three different replicates ± SE.

Treatments			Ca (mg/100 g DW)	Zn (mg/100 g DW)	K (mg/100 g DW)	P (mg/100 g DW)
Normal season	Shandaweel 1	Control	312 ± 0.333 ^c^	5.4 ± 0.033 ^b^	276 ± 0.333 ^l^	13.2 ± 0.088 ^a^
		1.5 mM silicon	352 ± 0.577 ^f^	7.32 ± 0.058 ^c^	216 ± 0.666 ^k^	36.0 ± 0.240 ^e^
		1.66 mM nanosilicon	348 ± 2.00 ^e^	7.45 ± 0.028 ^c^	198 ± 0.33 ^j^	48.0 ± 0.240 ^g^
	Gemmeiza 9	Control	264 ± 0.333 ^a^	4.44 ± 0.127 ^a^	192 ± 0.66 ^i^	19.2 ± 0.057 ^b^
		1.5 mM silicon	372 ± 0.577 ^g^	5.52 ± 0.058 ^b^	182 ± 0.33 ^h^	30.0 ± 0.115 ^d^
		1.66 mM nanosilicon	288 ± 0.333 ^b^	4.68 ± 0.037 ^a^	174 ± 0.33 ^g^	24.4 ± 0.202 ^c^
Late season	Shandaweel 1	Control	414 ± 1.00 ^h^	9.72 ± 0.151 ^d^	51.6 ± 0.29 ^f^	44.4 ± 0.272 ^f^
		1.5 mM silicon	489.6 ± 0.290 ^j^	11.52 ± 0.037 ^f^	40.8 ± 0.208 ^d^	148.8 ± 0.152 ^k^
		1.66 mM nanosilicon	423.2 ± 0.305 ^i^	10.8 ± 0.185 ^e^	37.2 ± 0.115 ^b^	141.6 ± 0.088 ^j^
	Gemmeiza 9	Control	310.8 ± 0.305 ^c^	7.2 ± 0.057 ^c^	43.2 ± 0.176 ^e^	63.6 ± 0.145 ^i^
		1.5 mM silicon	567.7 ± 0.493 ^k^	11.28 ± 0.188 ^f^	34.8 ± 0.233 ^a^	92.4 ± 0.768 ^i^
		1.66 mM nanosilicon	327.2 ± 0.635 ^d^	9.65 ± 0.060 ^d^	38.4 ± 0.152 ^c^	72.0 ± 1.083 ^h^

Values with different letters in column are significantly different at *p* < 0.05.

**Table 6:** Effects of silicon and nanosilicon on inorganic phosphorous, phytate P, phytic acid and oxalic acid contents in grains of two wheat cv. Shandaweel 1 and cv. Gemmeiza 9 at normal and late seasons. Each value is the main of three different replicates ± SE.

Treatments			Inorganic P (mg/100 g DW)	Phytate P (mg/100 g DW)	Phytic Acid (mg/100 g DW)	Oxalic Acid (mg/g DW)
Normal season	Shandaweel 1	Control	4.8 ± 0.088 ^a^	47.9 ± 0.264 ^h^	170.0 ± 0.088 ^i^	2.20 ± 0.057 ^a,b^
		1.5 mM silicon	11.2 ± 0.033 ^d^	41.6 ± 0.173 ^c^	147.6 ± 0.100 ^c^	2.64 ± 0.003 ^a,b^
		1.66 mM nanosilicon	15.6 ± 0.218 ^f^	41.8 ± 0.284 ^c^	148.3 ± 0.200 ^d^	1.76 ± 0.008 ^a,b^
	Gemmeiza 9	Control	6.0 ± 0.033 ^b^	57.5 ± 0.185 ^i^	204.1 ± 0.145 ^j^	3.08 ± 0.015 ^a,b^
		1.5 mM silicon	9.6 ± 0.033 ^c^	42.6 ± 0.218 ^d^	151.2 ± 0.057 ^e^	1.76 ± 0.015 ^a,b^
		1.66 mM nanosilicon	14.8 ± 0.185 ^e^	41.8 ± 0.240 ^c^	148.3 ± 0.100 ^d^	1.32 ± 0.025 ^a^
Late season	Shandaweel 1	Control	14.4 ± 0.057 ^e^	39.1 ± 0.120 ^b^	138.8 ± 0.088 ^b^	1.32 ± 0.031 ^a^
		1.5 mM silicon	48.0 ± 0.218 ^k^	43.8 ± 0.233 ^e^	155.4 ± 0.033 ^f^	0.88 ± 0.015 ^a^
		1.66 mM nanosilicon	46.8 ± 0.152 ^j^	44.4 ± 0.115 ^f^	157.6 ± 0.173 ^g^	1.02 ± 0.003 ^a^
	Gemmeiza 9	Control	31.2 ± 0.200 ^i^	33.1 ± 0.185 ^h^	117.5 ± 0.088 ^a^	1.76 ± 0.003 ^a,b^
		1.5 mM silicon	30.0 ± 0.218 ^h^	44.5 ± 0.185 ^f^	157.9 ± 0.173 ^g^	1.32 ± 4.026 ^b^
		1.66 mM nanosilicon	24.0 ± 0.251 ^g^	45.5 ± 0.208 ^g^	161.5 ± 0.088 ^h^	0.88 ± 0.003 ^a^

Values with different letters in column are significantly different at *p* < 0.05.

**Table 7:** Effects of silicon and nanosilicon on the straw yield quality of two wheat cv. Shandaweel 1 and cv. Gemmeiza 9 at normal and late seasons. Each value is the main of three different replicates ± SE.

Treatments			Straw Yield (g/Plant)	Soluble Sugar (mg/g DW)	Insoluble Sugar (mg/g DW)	Starch (mg/g DW)	α-Amylase (mg Sugar/Mmin./g DW)
Normal season	Shandaweel 1	Control	34.1 ± 0.014 ^c^	4.52 ± 0.093 ^f^	17.6 ± 0.088	7.71 ± 0.201 ^d^	53.0 ± 0.033 ^h^
		1.5 mM silicon	41.6 ± 0.035 ^e^	6.63 ± 0.095 ^d^	14.4 ± 0.088	0.96 ± 0.012 ^a^	72.8 ± 0.166 ^k^
		1.66 mM nanosilicon	39.9 ± 0.096 ^i^	5.83 ± 0.159 ^e^	16.9 ± 0.218	0.96 ± 0.025 ^a^	69.5 ± 0.088 ^j^
	Gemmeiza 9	Control	27.7 ± 0.095 ^f^	4.62 ± 0.121 ^h^	25.3 ± 0.066	7.23 ± 0.029 ^d^	50.1 ± 0.088 ^e^
		1.5 mM silicon	39.6 ± 0.061 ^h^	5.54 ± 0.042 ^a^	9.6 ± 0.218	4.34 ± 0.059 ^b^	52.1 ± 0.057 ^g^
		1.66 mM nanosilicon	36.5 ± 0.159 ^j^	8.91 ± 0.242 ^a^	9.0 ± 0.120	5.78 ± 0.030 ^c^	54.8 ± 0.057 ^i^
Late season	Shandaweel 1	Control	27.7 ± 0.076 ^b^	5.71 ± 0.176 ^e^	16.9 ± 0.166	16.21 ± 0.065 ^f^	49.8 ± 0.166 ^d^
		1.5 mM silicon	32.6 ± 0.135 ^d^	4.08 ± 0.031 ^c^	11.6 ± 0.264	24.58 ± 0.126 ^h^	42.6 ± 0.033 ^b^
		1.66 mM nanosilicon	30.7 ± 0.165 ^g^	4.55 ± 0.125 ^b^	10.9 ± 0.088	23.14 ± 0.117 ^g^	39.3 ± 0.120 ^a^
	Gemmeiza 9	Control	19.7 ± 0.213 ^a^	7.14 ± 0.078 ^g^	19.5 ± 0.066	13.37 ± 0.095 ^e^	48.8 ± 0.152 ^f^
		1.5 mM silicon	32.2 ± 0.161 ^d^	5.04 ± 0.029 ^c^	11.8 ± 0.153	33.26 ± 0.107 ^j^	39.6 ± 0.202 ^a^
		1.66 mM nanosilicon	34.1 ± 0.014 ^c^	4.52 ± 0.093 ^f^	17.6 ± 0.088	7.71 ± 0.201 ^d^	53.0 ± 0.033 ^h^

Values with different letters in column are significantly different at *p* < 0.05.

**Table 8:** Effects of silicon and nanosilicon on the straw yield quality of two wheat cv. Shandaweel 1 and cv. Gemmeiza 9 at normal and late seasons. Each value is the main of three different replicates ± SE.

Treatments			Soluble Protein (mg/g DW)	Insoluble Protein (mg/g DW)	Total Phenols (mg/g DW)	Flavonoids (mg/g DW)	Total Antioxidant Capacity (µg/g DW)
Normal season	Shandaweel 1	Control	1.67 ± 0.031 ^c^	3.75 ± 0.008 ^i^	1.92 ±0.008 ^a^	0.52 ± 0.033 ^a^	0.29 ± 0.015 ^e^
		1.5 mM silicon	2.41 ± 0.006 ^f^	2.21 ± 0.006 ^d^	3.07 ± 0.017 ^c^	0.61 ± 0.011 ^c^	0.16 ± 0.002 ^b^
		1.66 mM nanosilicon	2.29 ± 0.008 ^e^	2.33 ± 0.010 ^e^	3.17 ± 0.010 ^d^	0.57 ± 0.006 ^b^	0.16 ± 0.001 ^b^
	Gemmeiza 9	Control	1.58 ± 0.012 ^a^	3.65 ± 0.005 ^h^	3.25 ± 0.008 ^e^	0.59 ± 0.003 ^b,c^	0.12 ± 0.003 ^a^
		1.5 mM silicon	2.21 ± 0.003 ^d^	2.17 ± 0.005 ^c^	3.72 ± 0.011 ^h^	0.92 ± 0.017 ^e^	0.19 ± 0.004 ^c^
		1.66 mM nanosilicon	2.63 ± 0.013 ^g^	2.21 ± 0.003 ^d^	3.62 ± 0.005 ^g^	1.17 ± 0.015 ^h^	0.16 ± 0.008 ^b^
Late season	Shandaweel 1	Control	2.17 ± 0.030 ^b^	3.34 ± 0.005 ^g^	2.74 ± 0.003 ^b^	0.85 ± 0.014 ^d^	0.56 ± 0.013 ^f^
		1.5 mM silicon	3.00 ± 0.005 ^h^	2.08 ± 0.016 ^b^	5.37 ± 0.014 ^l^	1.05 ± 0.014 ^f^	0.18 ± 0.001 ^c^
		1.66 mM nanosilicon	3.67 ± 0.063 ^k^	2.17 ± 0.005 ^c^	3.57 ± 0.003 ^f^	1.43 ± 0.008 ^i^	0.19 ± 0.002 ^c^
	Gemmeiza 9	Control	2.21 ± 0.003 ^d^	3.27 ± 0.008 ^f^	3.84 ± 0.008 ^i^	1.11 ± 0.008 ^g^	0.64 ± 0.013 ^g^
		1.5 mM silicon	3.25 ± 0.006 ^i^	2.04 ± 0.014 ^b^	3.98 ± 0.028 ^j^	1.92 ± 0.027 ^k^	0.25 ± 0.007 ^d^
		1.66 mM nanosilicon	1.67 ± 0.031 ^c^	3.75 ± 0.008 ^i^	1.92 ± 0.008 ^a^	0.52 ± 0.033 ^a^	0.29 ± 0.015 ^e^

Values with different letters in column are significantly different at *p* < 0.05.

**Table 9:** Effects of silicon and nanosilicon on inorganic phosphorous, phytate P, phytic acid and oxalic acid contents in straw of two wheat cv. Shandaweel 1 and cv. Gemmeiza 9 at normal and late seasons. Each value is the main of three different replicates ± SE.

Treatments			Inorganic P (mg/100 g DW)	Phytate P (mg/100 g DW)	Phytic Acid (mg/100 g DW)	Oxalic Acid (mg/g DW)
Normal season	Shandaweel 1	Control	37.2 ± 0.115 ^i^	41.04 ± 0.127 ^d^	145.6 ± 0.202 ^c,d^	2.20 ± 0.057 ^e^
		1.5 mM silicon	27.6 ± 0.115 ^f^	44.3 ± 0.10 ^a^	157.2 ± 0.633 ^f,g^	3.52 ± 0.003 ^g^
		1.66 mM nanosilicon	33.6 ± 0.066 ^h^	43.76 ± 0.046 ^b^	155.3 ± 0.133 ^f^	2.20 ± 0.033 ^e^
	Gemmeiza 9	Control	40.8 ± 0.152 ^j^	40.1 ± 0.033 ^c^	142.3 ± 1.874 ^c^	2.64 ± 0.003 ^f^
		1.5 mM silicon	37.2 ± 0.115 ^i^	43.52 ± 0.006 ^b^	154.4 ± 0.145 ^f^	2.20 ± 0.057 ^e^
		1.66 mM nanosilicon	28.8 ± 0.057 ^g^	44.94 ± 0.173 ^b^	159.5 ± 0.735 ^g^	1.76 ± 0.003 ^c^
Late season	Shandaweel 1	Control	26.4 ± 0.669 ^e^	35.52 ± 0.103 ^c^	126.1 ± 1.102 ^a^	1.32 ± 0.006 ^a^
		1.5 mM silicon	13.2 ± 0.088 ^d^	42.0 ± 0.033 ^b^	149.1 ± 0.698 ^e^	1.76 ± 0.030 ^c^
		1.66 mM nanosilicon	7.2 ± 0.115 ^b^	41.94 ± 0.173 ^c^	148.8 ± 0.517 ^d,e^	1.54 ± 0.003 ^b^
	Gemmeiza 9	Control	13.2 ± 0.115 ^d^	36.76 ± 0.046 ^d^	130.4 ± 0.854 ^b^	1.32 ± 0.028 ^a^
		1.5 mM silicon	2.4 ± 0.066 ^a^	41.4 ± 0.033 ^c^	146.9 ± 1.047 ^de^	1.72 ± 0.031 ^a^
		1.66 mM nanosilicon	12.0 ± 0.033 ^c^	41.28 ± 0.052 ^b^	146.5 ± 0088 ^c,d,e^	1.98 ± 0.015 ^d^

Values with different letters in column are significantly different at *p* < 0.05.

**Table 10:** Effects of silicon and nanosilicon on calcium (Ca), zinc (Zn), phosphorous (P) and potassium (K) in straw of two wheat cv. Shandaweel 1 and cv. Gemmeiza 9 at normal and late seasons. Each value is the main of three different replicates ± SE.

Treatments			Ca (mg/100 g DW)	Zn (mg/100 g DW)	P (mg/100 g DW)	K (mg/100 g DW)
Normal season	Shandaweel 1	Control	176 ± 0.33 ^c^	24.0 ± 0.033 ^j^	34.8 ± 0.033 ^b^	112.8 ± 0.088 ^g^
		1.5 mM silicon	185 ± 0.33 ^e^	9.72 ± 0.072 ^f^	40.6 ± 0.033 ^d^	85.2 ± 0.115 ^d^
		1.66 mM nanosilicon	180 ± 0.33 ^d^	12.0 ± 0.033 ^h^	51.9 ± 0.033 ^e^	100.8 ± 3.153 ^f^
	Gemmeiza 9	Control	132 ± 0.33 ^a^	10.92 ± 0.070 ^g^	30.0 ± 0.033 ^a^	124.8 ± 0.057 ^h^
		1.5 mM silicon	132 ± 0.66 ^a^	9.96 ± 0.111 ^f^	37.2 ± 0.033 ^c^	115.2 ± 0.057 ^g^
		1.66 mM nanosilicon	144 ± 0.33 ^b^	9.84 ± 0.140 ^f^	40.7 ± 0.033 ^d^	88.8 ± 0.152 ^e^
Late season	Shandaweel 1	Control	228 ± 0.33 ^h^	12.84 ± 0.140 ^i^	144 ± 0.33 ^f^	82.8 ± 0.145 ^d^
		1.5 mM silicon	360 ± 0.33 ^k^	7.2 ± 0.033 ^d^	168 ± 0.33 ^h^	40.8 ± 0.057 ^c^
		1.66 mM nanosilicon	292 ± 0.33 ^j^	5.16 ± 0.157 ^c^	150 ± 0.33 ^g^	21.6 ± 0.145 ^a^
	Gemmeiza 9	Control	216 ± 0.33 ^f^	7.68 ± 0.058 ^e^	150 ± 0.66 ^g^	84.0 ± 0.088 ^d^
		1.5 mM silicon	220 ± 0.33 ^g^	3.48 ± 0.122 ^b^	180.4 ± 0.115 ^i^	37.2 ± 0.088 ^b^
		1.66 mM nanosilicon	252 ± 0.33 ^i^	2.61 ± 0.068 ^a^	186 ± 0.66 ^j^	40.8 ± 0.088 ^c^

Values with different letters in column are significantly different at *p* < 0.05.

## Data Availability

The data presented in this study are available in [insert article].
