# Correction: Helal et al. Improving Yield Components and Desirable Eating Quality of Two Wheat Genotypes Using Si and NanoSi Particles under Heat Stress. *Plants* 2022, *11*, 1819

**DOI:** 10.3390/plants12142637

**Published:** 2023-07-13

**Authors:** Nesma M. Helal, Hemmat I. Khattab, Manal M. Emam, Gniewko Niedbała, Tomasz Wojciechowski, Inès Hammami, Nadiyah M. Alabdallah, Doaa Bahaa Eldin Darwish, Mohamed M. El-Mogy, Heba M. Hassan

**Affiliations:** 1 Botany Department, Faculty of Science, Ain Shams University, Cairo 11566, Egypt; nesmaflax@yahoo.co.uk (N.M.H.); dr.hemmat@hotmail.com (H.I.K.); emammanal@ymail.com (M.M.E.); 2 Department of Biosystems Engineering, Faculty of Environmental and Mechanical Engineering, Poznań University of Life Sciences, Wojska Polskiego 50, 60-627 Poznań, Poland; tomasz.wojciechowski@up.poznan.pl; 3 Department of Biology, College of Science, Imam Abdulrahman Bin Faisal University, P.O. Box 1982, Dammam 31441, Saudi Arabia; ihammami@iau.edu.sa (I.H.); nmalabdallah@iau.edu.sa (N.M.A.); 4 Botany Department, Faculty of Science, Mansoura University, Mansoura 35511, Egypt; ddarwish@ut.edu.sa; 5 Biology Department, Faculty of Science, University of Tabuk, Tabuk 46429, Saudi Arabia; 6 Vegetable Crops Department, Faculty of Agriculture, Cairo University, Giza 12613, Egypt; elmogy@agr.cu.edu.eg

In the original publication [1], there was a mistake inure 1 as published. Figure 1a was accidentally duplicated in the original article. The corrected Figure 1 appears below. 


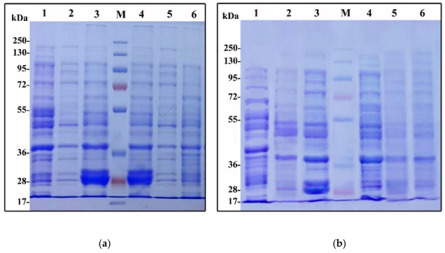


The authors state that the scientific conclusions are unaffected. This correction was approved by the Academic Editor. The original publication has also been updated.
